# Bacterial Profile and Antibiotic Resistance in Oral and Maxillofacial Infections

**DOI:** 10.3390/jcm15124642

**Published:** 2026-06-15

**Authors:** Michał Lenart, Maciej Sikora, Maciej Okła, Łukasz Słowik, Katarzyna Błochowiak

**Affiliations:** 1 Department of Maxillofacial Surgery, Hospital of the Ministry of Interior, 25-375 Kielce, Poland; coxalgan1@gmail.com (M.L.); sikora-maciej@wp.pl (M.S.); 2 Department of Biochemistry and Medical Chemistry, Pomeranian Medical University, 70-111 Szczecin, Poland; 3 National Medical Institute of the Ministry of Interior and Administration, 02-507 Warsaw, Poland; 4 Department of Maxillofacial Surgery, Poznan University of Medical Sciences, 60-355 Poznan, Poland; maciejokla@ump.edu.pl (M.O.); lukasz.slowik@ump.edu.pl (Ł.S.); 5 Department of Oral Surgery, Poznan University of Medical Sciences, 60-812 Poznan, Poland

**Keywords:** odontogenic infections, antibiotic resistance, antibiotics, deep neck infections, maxillofacial infections, anaerobic bacteria, aerobic bacteria, drug resistance

## Abstract

**Background/Objectives**: Oral and maxillofacial infections present polybacterial profiles, including both aerobic and anaerobic bacteria. Increasing antibiotic resistance poses a significant challenge to pharmacological treatment of these infections. The aim of this study was to present a bacterial profile and assess antibiotic resistance found in these infections. **Methods**: This retrospective analysis is based on medical records of 224 patients affected with maxillofacial infections. Microbiological cultures and antibiotic susceptibility testing were performed for all patients. **Results**: In 78.57% of the patients, a positive microbiological culture was obtained. A total of 72.72% of culture-positive patients showed multi-bacterial cultures (128/176). Predominant bacteria included *Streptococcus*, detected in 156 cases (39%), followed by *Staphylococcus*, found in 64 cases (16%), and *Prevotella*, detected in 56 of 400 total bacterial isolates (14%). The most often isolated aerobic strains were *Streptococcus mitis/oralis* detected in 64 (16%) cases and *Staphylococcus epidermidis* detected in 48 cases (12%), while the most common anaerobic strains were *Prevotella buccae* detected in 14 cases (3.5%). *Streptococcus* and *Staphylococcus* exhibited the greatest resistance to clindamycin, accounting for 51.74% and 47.63%, respectively. Aerobic Gram-positive cocci were more resistant to penicillin and amoxicillin than to cephalosporins. Among obligate anaerobes, the lowest antibiotic resistance seen was to metronidazole. The obligate anaerobes except *Prevotella* were sensitive to clindamycin. **Conclusions**: A high rate of clindamycin resistance among aerobic and facultatively anaerobic Gram-positive cocci indicates the need to reassess the use of clindamycin in empirical therapy. The bacterial composition of infections suggests the need to use combined antibiotic therapy. First- and second-generation cephalosporins may be an effective alternative to penicillin and its derivatives.

## 1. Introduction

Infections of the head and neck are among the most common reasons for patients’ hospitalization and outpatient treatment in maxillofacial surgery. It is estimated that up to 90% of them result from odontogenic causes, including severe periapical periodontitis and pericoronitis. Numerous odontogenic infections are self-limiting and could be effectively treated surgically, together with implementing causative treatment. However, some can spread to adjacent fascial spaces and thus require more antibacterial therapy, especially in compromised patients [1]. The most severe maxillofacial infections may result in systemic and local complications, including sepsis, phlegmon, osteomyelitis, mediastinitis and airway-related complications, including pneumonia and airway obstruction. The likelihood of complications is increased by patient self-medication, inadequate clinical management, severe and uncontrolled systemic diseases, and bacterial resistance. Increased mortality in maxillofacial infections and the risk of serious complications are brought about by antibiotic overuse and replacing surgical treatment with pharmacological treatment [2]. It is estimated that 10% of all antibiotics used in such treatment are prescribed by dentists, and 55–80% of them could be considered overprescribed [1]. This practice contributes to higher rates of hospitalization due to odontogenic infections and the choice of resistant strains. Antibiotic resistance is a significant burden on healthcare providers and increases the mortality rate of head and neck infections from 10% to 40%, especially in patients with multiple morbidities [2]. Many bacterial species previously successfully treated with antibiotics have lost sensitivity because of indiscriminate use of the most common antibiotics and routine implementation of empirical antibiotic therapy, even in maxillofacial infections treated in outpatient surgeries [3]. Implementation of empirical antibiotic therapy before pathogen identification is carried out is a widespread practice and is due to the delay in microbial culture and susceptibility testing. Another problem in antibiotic therapy is widespread empirical labeling of penicillin hypersensitivities based on nonspecific or misinterpreted symptoms. It often leads to the application of less effective antibiotics and the need to replace penicillin with broader-spectrum antibiotics, thus contributing to developing resistance.

Furthermore, some previously applied antibiotic schemes could become clinically ineffective due to changes in bacterial distribution and might require new recommendations. One of the main challenges in pharmacological therapy of odontogenic infections is the polymicrobial nature of these infections [4,5]. Such character of these infections often requires a combination of various antibiotics effective on aerobic as well as anaerobic bacteria [4,5]. Some maxillofacial infections may be characterized by the presence of untypical bacteria such as *Pseudomonas aeruginosa*, *Klebsiella pneumoniae*, *Escherichia coli*, *Acinetobacter ursingii*, and *Serratia marcescens*. A higher percentage of these bacteria in the maxillofacial infections can be associated with alcohol use disorder, diabetes mellitus and immunocompromised patients. They can predispose one to a more severe course of these infections and deep neck space involvement. They do not usually occur in pediatric patients [6,7]. Furthermore, possible changes in the maxillofacial infections, bacterial composition and bacterial resistance to antibiotics could be ascribed to the COVID-19 pandemic. The pandemic significantly limited access to hospital and outpatient treatment for patients suffering from odontogenic infections. Moreover, in the COVID-19 pandemic, antibiotics were used on a massive scale and altered empirical prescribing. Differences in retrospective studies compared to those from the pre-COVID-19 era possibly reflect this phenomenon. Previous studies that assessed the impact of the pandemic on the course of maxillofacial infections focused on variations in their clinical course, including hospital stay duration, the level of inflammatory markers, the number of involved fascial spaces, and the general severity of odontogenic infections [8,9]. However, data on its impact on antibiotic resistance and bacterial composition are limited. Our previous study mainly presented the results obtained in the pre-pandemic and early pandemic periods [6,7]. In our opinion, it is justified to supplement these data. Moreover, empirical antibiotic regimens do not take into account population differences and the profile of maxillofacial infections in different countries. Therefore, from the clinical point of view, it is important to elicit and present the most current and population-diverse data related to maxillofacial infections and bacterial resistance. We believe contemporary data on bacterial profiles and antibiotic resistance patterns specific to Central European populations remain scarce, and the data from the COVID-19/post-COVID-19 era are largely absent [1,10]. This study can address that gap. Only comprehensive and up-to-date data are helpful in setting up indications for pharmacological treatment of maxillofacial infections, the choice of a suitable antibiotic, and the knowledge of microorganisms responsible for these infections. The aim of this study was to analyze the current bacterial profile of maxillofacial infections and the antibiotic resistance of the most often identified bacterial species.

## 2. Materials and Methods

We reviewed medical records of 224 patients with a diagnosis of maxillofacial infections treated in the Department of Maxillofacial Surgery of the Poznan University of Medical Sciences in Poznan and in the Department of Maxillofacial Surgery of the Hospital of the Ministry of Interior in Kielce from January 2022 to December 2025. A total of 64 patients were treated in 2022–2023, and 160 patients were treated in 2024–2025. Recorded data included demography, etiology, symptoms and site of infection, treatment and hospital care details, duration of infection, complications, identified bacterial species, accompanying systemic diseases, and antibiotics used to fight infections. Bacteriological examinations with antibiogram and drug resistance assessment were carried out. Exclusion criteria included head and neck tumors and superficial skin abscesses. All material for microbiological analysis was collected during surgery and drainage. Both aerobic and anaerobic bacterial cultures were performed using an aseptic technique. Samples were collected using the DeltaSwab Amies kit (Deltalab), which features a sterile swab with Amies gel transport medium. According to the manufacturer’s specifications, validated in accordance with the CLSI M40-A2 standard, this medium maintains the viability of aerobic, facultative anaerobic, and obligate anaerobic microorganisms for at least 48 h at both room temperature (20–25 °C) and refrigeration temperature (2–8 °C). The material was delivered to the laboratory within 2 h of collection (<2 h), further minimizing the risk of viability loss for anaerobic microorganisms. The following bioMérieux media were used for anaerobic culture:

— Schaedler Agar: a non-selective medium for the cultivation of fastidious anaerobes.
— Schaedler Neo. Vanco. Agar + 5% sheep blood (SNVS): a selective medium containing neomycin and vancomycin, supplemented with 5% sheep blood.
— Schaedler KV Agar: a selective medium with kanamycin and vancomycin for the isolation of Gram-negative anaerobes.
— Schaedler Broth with 0.02% Agar + vit. K3: a liquid enrichment medium supplemented with vitamin K3. Solid media were incubated under strictly anaerobic conditions at 35–37 °C using the GENbag anaer pouch system (bioMérieux). The initial growth assessment on solid media and in the anaerobic broth was performed after 48 h. In the event of a negative result, broth incubation was extended up to 7 days before the final evaluation. This protocol enabled the detection of slow-growing anaerobes. Antimicrobial susceptibility was determined using the disk diffusion method and/or the gradient method (E-test) with strips containing a minimum inhibitory concentration (MIC) gradient. The choice of method depended on the isolated microorganism species. Results were interpreted in accordance with the EUCAST guidelines applicable at the time of this study.

Empirical antimicrobial therapy was implemented as the first choice, and the treatment was modified in chosen cases to match the results of microbiological analysis. Surgical treatment included incision and drainage of abscesses under local or general anesthesia.

This study was performed in accordance with the ethical standards laid down in an appropriate version of the World Medical Association Declaration of Helsinki. According to the recommendations of the Bioethics Committee of the Poznan University of Medical Sciences, retrospective studies based on one’s own medical records do not require the consent of the committee. According to Polish law and Good Clinical Practice (GCP) regulations, the scientific research entitled “Bacterial profile and antibiotic resistance in oral and maxillofacial infections” does not require the approval of the Bioethics Committee at the Poznan University of Medical Sciences. The Bioethics Committee at the Poznan University of Medical Sciences confirmed that this research is not a medical experiment (KB-309/26) on 22 April 2026.

The calculations were carried out with Microsoft Excel 2016 and STATISTICA software (v.13 TIBCO, Palo Alto, CA, USA). Categorical variables were presented in contingency tables, and their associations were tested, depending on the number of cases, with Fisher–Freeman–Halton’s test, Fisher’s exact test or Chi^2^ Pearson’s test. Bonferroni’s correction was used only for multiple comparisons of antibiotic resistance between bacterial species grouped according to the bacterial phylum and types. *p* < 0.05 was considered statistically significant.

## 3. Results

### 3.1. Characteristics of the Study Group

Summarized patients’ demographic and clinical characteristics are presented in Table 1. Predominant antibiotic treatment included two antibiotics. In 42 patients, only one antibiotic was used. In two patients, a third antibiotic was added. The detailed data regarding antibiotic use is presented in Table 1.

### 3.2. Bacterial Profile of Maxillofacial Infections

Among a total number of 224 patients, in 176 patients (78.57%), a positive microbiological culture was obtained. Single bacterial strains were isolated in 48 patients, accounting for 27.27%. Multi-bacterial cultures were detected in 128 cases, accounting for 72.72%. The predominant bacteria included *Streptococcus*, detected in 156 cases (39%), followed by *Staphylococcus*, identified in 64 cases (16%), and *Prevotella*, detected in 56 cases (14%). The most often isolated aerobic strains were *Streptococcus mitis/oralis* found in 64 (16%) cases and *Staphylococcus epidermidis* in 48 cases (12%). The most common anaerobic strain was *Prevotella buccae*, detected in 14 cases (3.5%). Aerobic and relative anaerobic bacteria were found 276 times, accounting for 69%, whereas obligate anaerobic bacteria were found 124 times, accounting for 31%. The ratio of aerobic and relative anaerobic bacteria to obligate anaerobic bacteria was 2:1. Among aerobic bacteria *Streptococcus* predominated, followed by *Staphylococcus*. Among anaerobic bacteria Gram-negative bacilli, including *Prevotella* and *Fusobacterium*, predominated. In 11 patients, only obligate anaerobic bacteria were detected. In 66 patients, isolated bacterial culture included both aerobic/relative anaerobic bacterial species and obligate anaerobic bacterial species.

Detailed distribution of cultured bacterial species is presented in Table 2.

Excluding bacterial taxonomy, the most commonly identified bacterial groups included aerobic and relatively anaerobic bacteria classified as Gram-positive cocci such as *Streptococcus* and *Staphylococcus*, detected in 220 cases. Among obligate anaerobic bacteria, the most commonly identified bacteria were Gram-negative bacilli (*Prevotella*), Gram-negative cocci (*Veillonella*), and *Fusobacterium,* detected in 55, 16, and 16 cases, respectively.

### 3.3. Antibiotic Resistance in Aerobic and Facultative Anaerobic Bacterial Species

Among the most common identified bacteria, both *Streptococcus* and *Staphylococcus* exhibited the greatest resistance to clindamycin. Considering β-lactam antibiotics, *Streptococcus* was more resistant to penicillin than to cephalosporins. The antibiotic most effective on *Streptococcus*, *Staphylococcus* and *Enterococcus* was vancomycin. Detailed assessment of antibiotic resistance in the group of aerobic and facultative anaerobic bacteria is presented in Table 3.

### 3.4. Antibiotic Resistance in Obligate and Facultative Anaerobic Bacterial Species

Obligate anaerobic bacteria showed the lowest resistance to metronidazole. Obligate anaerobic bacteria, except *Prevotella*, were sensitive to clindamycin. *Fusobacterium* showed low resistance to clindamycin. The anaerobic *Schaalia* and *Lancefieldella* were resistant to clindamycin and were sensitive to penicillin, as presented in Table 4.

### 3.5. Comparing Antibiotic Resistance Between Aerobic and Anaerobic Bacteria

Obligate anaerobic bacterial species showed high resistance to penicillin and amoxicillin. There is a statistically significant difference in resistance to penicillin and amoxicillin between aerobic or facultative bacteria and obligate anaerobic bacteria **(*p* = 0.003** and ***p* = 0.000),** as shown in Table 5.

### 3.6. Comparing Antibiotic Resistance Between Bacterial Species Grouped According to the Bacterial Phylum and Types

Thorough statistical analysis with Bonferroni’s correction showed a statistically significant difference in resistance to clindamycin between Firmicutes, including mainly Gram-positive aerobic *Streptococcus*, and Fusobacteriota, represented mainly by anaerobic Gram-negative *Fusobacterium* **(*p* = 0.025).** There was a statistically significant difference in resistance to Sulfamethoxazole/Trimethoprim between Firmicutes and Proteobacteria **(*p* = 0.010),** as shown in Table 6. All isolates belonging to Proteobacteria exhibited resistance to clindamycin.

Bacteria of the Bacillota group, represented mainly by Gram-positive aerobic cocci and Gram-negative anaerobic cocci, showed statistically significant lower resistance to amoxicillin and penicillin compared to Bacteroidota, represented mainly by Gram-negative anaerobic bacilli such as *Prevotella* **(*p* = 0.000)**. Actinomycetota, represented by *Schaalia* and *Rothia*, showed higher resistance to metronidazole compared to Bacteroidota, represented by Gram-negative anaerobic bacilli, including *Prevotella* (*p* = 0.057) in Table 7.

## 4. Discussion

Antibacterial drug resistance is a serious clinical problem and a burden on the healthcare system. By implementing pharmacological treatment for odontogenic infections, among others, dentists can affect proper drug policy and reduce the incidence of antibiotic resistance. Therefore, the aim of this study was to assess the resistance of the bacterial strains most frequently occurring in oral and maxillofacial infections to the antibiotics used in their treatment. The results obtained may help in developing an effective regimen of empirical antibiotic therapy in these infections that will consider their bacteriological profile and antibiotic resistance patterns.

Our findings revealed that odontogenic infections possess a specific bacterial profile. The percentage distribution of aerobic and anaerobic bacteria and the predominance of aerobic Gram-positive cocci found in our study are consistent with the previous findings [6,7,10,11,12,13]. It reflects the polymicrobial nature of odontogenic infections and a relatively great proportion of anaerobic bacteria. The bacterial species detected in our study build oral flora normally found in the mouth and could be causative pathogens of odontogenic infections. As emphasized in our previous study, odontogenic infections have a specific clinical course. Their specific pattern from diffuse and painful cellulitis to the more localized and painless abscesses explained the shift of the bacterial content from aerobic bacterial species to anaerobic bacterial species as the amount of oxygen decreases. Aerobic bacteria predominate in the initial stage of odontogenic infections and decrease in favor of anaerobic bacteria in the more advanced stages [6,7]. However, the real proportion of anaerobic bacteria may be significantly underestimated due to the limitations of diagnostic methods. Furthermore, some authors indicate that anaerobic bacteria are the dominant microorganisms [14,15]. In the study conducted by Thoel et al., where next-generation sequencing was applied, anaerobes were found in 50 swabs, while aerobic bacteria were detected in 30. Four of the five most common bacterial genera were anaerobes, including *Fusobacterium*, *Prevotella*, *Parvimonas*, and *Porphyromonas* [14]. Earlier antibiotic therapy reduced aerobic bacteria identification frequency [14]. Some researchers noticed other factors that could affect changes in bacterial distribution, especially among the same type of bacteria [16]. They included age, concomitant diseases, and the cause of infection and were effective in producing differences in bacterial composition of odontogenic infections. Obesity, older age and some systemic diseases, including diabetes, may promote different bacterial species, even among the predominant aerobic cocci. Moreover, differences in bacterial composition toward a higher proportion of *Staphylococcus* in inpatient odontogenic infections, compared to outpatient infections, were present [12]. Although odontogenic infections result from similar bacteria, there are discrepancies in bacterial distribution between patients with severe periodontitis and those who complain of periapical periodontitis or pericoronitis [17]. Brescó Salinas et al. found that lower third molar pericoronitis was mainly attributed to Gram-positive facultative anaerobic cocci of the *Streptococcus* genus, followed by Gram-negative strict anaerobes *Fusobacterium* and *Prevotell,* compared to periapical lesions [17]. Final bacterial composition could be reflected by the primary origin of the infection, including the upper respiratory tract and gastrointestinal tract. This relationship was reported in the studies where odontogenic infections were compared with non-odontogenic ones. Non-odontogenic ones showed a relatively higher proportion of *Staphylococcus aureus*, *Staphylococcus epidermidis*, and *Streptococcus viridans*, as well as Gram-negative bacteria *Pseudomonas aeruginosa*, *Escherichia coli*, *and Klebsiella pneumoniae*, than odontogenic infections [7,18,19]. Moreover, oral hygiene, oral habits, long-term nicotine or alcohol abuse and lifestyle can predispose one to these dissimilarities [6,18]. Involvement of fascial space is very likely to change the bacterial profile of odontogenic infections, too. Submandibular space is the most frequently affected fascial space in odontogenic infections, which was also confirmed by our study. However, a shift in bacterial composition of odontogenic infections may be linked to the involvement of other fascial spaces. Proximity to the lateral pharyngeal space and peritonsillar area in some infections may promote greater distribution of group *A streptococci* and the *Streptococcus milleri* group, especially *Streptococcus anginosus*, which are the most isolated aerobes in peritonsillar abscesses [19,20,21]. Finally, a relatively higher proportion of anaerobic bacteria such as *Prevotella* compared to *Fusobacterium* and *Porphyromonas* reported in other studies may be the effect of limitations in collecting the material for microbiological tests [22].

The resistance to antibiotics usually used in odontogenic infections was another issue that our study assessed. It is a serious obstacle to make the treatment effective. In the study by Thol et al., antibiotic resistance was detected in two-thirds of the swabs examined for resistance genes. These were most often directed against antibiotic substance classes of lincosamides, macrolides and tetracyclines [14]. Our study revealed a relatively high resistance of the predominant aerobic Gram-positive cocci, including *Streptococcus* and *Staphylococcus* to clindamycin. In our study, 51.74% of *Streptococcus* and 44.69% of Gram-positive cocci were resistant to clindamycin. It was much higher than resistance to amoxicillin, penicillin, amoxicillin + clavulanic acid and other β-lactam antibiotics such as cephalosporins. These results are consistent with those obtained in earlier studies [23,24,25]. This produces serious clinical implications because clindamycin is still widely used in the treatment of odontogenic infections and is shown as an effective antibiotic in combating aerobic Gram-positive cocci such as *Streptococci*. In addition, clindamycin is recommended as a first-line empirical antibiotic for odontogenic infections in patients with an allergy to penicillin [16,26,27]. It can be applied to fight bacterial invasion in the peri-implant area, reducing marginal bone loss [28]. It is considered an effective anti-anaerobic agent as well as active against *Streptococci* and methicillin-resistant *S. aureus*. Its bacterial spectrum includes nearly all the likely pathogens of odontogenic infections. Clindamycin gives good penetration into the jawbone and abscess cavities. However, these recommendations were based on the results obtained in older studies [13]. Some researchers underscore the rise in clindamycin prescribed by dentists [29]. So high resistance to clindamycin strongly suggests that its use should be critically reevaluated, particularly considering its limited suitability as first-line empirical therapy. However, microbiological resistance to clindamycin should be clearly distinguished from its potentially clinical failure when designing new recommendations for pharmacological treatment of maxillofacial infections. One of the possible reasons for a growing resistance to clindamycin is reported penicillin hypersensitivity. This has led to the excessive use of clindamycin and, as a result, to drug resistance. A direct relationship between resistance to clindamycin and penicillin hypersensitivity was confirmed in the study conducted by Fischer et al., who observed a significantly higher risk of resistance to clindamycin in patients with a documented penicillin hypersensitivity [22]. In most studies, penicillin and amoxicillin prove satisfactory efficacy against the most frequently isolated aerobic Gram-positive cocci, primarily *Streptococci*. No significant variations in resistance to penicillin and amoxicillin have been noticed over the past few years, and this resistance has been maintained at a steady level [13,23]. Moreover, it seems that the presence of clavulanic acid is not decidedly advantageous in the treatment of odontogenic infections. Bacteria isolated in odontogenic infections are more resistant to amoxicillin–clavulanic acid than to ceftriaxone. Ceftriaxone could be considered an empirical antibiotic for severe odontogenic infections instead of amoxicillin–clavulanic acid [30]. Seemingly, even the addition of clavulanic acid does not considerably increase the spectrum of antibacterial potential in odontogenic infections, which would reflect a rise in the treatment efficacy. Contrary to these findings, clavulanic acid can expand the spectrum to *Staphylococcus* and some anaerobes by conferring beta-lactamase resistance [31]. In our study, no substantial disparities in the resistance to amoxicillin, penicillin and amoxicillin combined with clavulanic acid in aerobic cocci were found. However, amoxicillin combined with clavulanic acid is more efficacious against *Veillonella* than amoxicillin without clavulanic acid. It seems that some cephalosporins could be successful equivalents of clavulanate. Moreover, cephalosporins are bactericidal and show few side effects. Some have broader antimicrobial spectra and show stronger bactericidal activity against the pathogens specific to orofacial odontogenic infections. In previous studies, cefazolin and cefmetazole were shown to exert great antimicrobial impact on *Streptococci*, *Peptostreptococcus*, *Porphyromonas*, and *Fusobacterium* [32]. Our study confirmed higher effectiveness of cephalosporins against aerobic Gram-positive cocci, especially *Streptococcus*, compared to amoxicillin alone and combined with clavulanic acid. Similarly, some earlier studies reported that the first and second generations of cephalosporins are efficacious against aerobic and anaerobic Gram-positive cocci, but their effect is unpredictable against anaerobic Gram-negative bacilli. Cefotaxime, which belongs to the third-generation cephalosporins, is highly potent against anaerobic bacteria, including mixed flora of dentoalveolar abscesses, and could be a particularly desirable choice in combating these infections. However, its efficacy against *Staphylococcus* and anaerobic bacterial species such as *Prevotella* and *Veillonella* should be assessed. In the study by Adamson et al., more bacteria isolated in maxillofacial patients were sensitive to ceftriaxone than to amoxicillin with clavulanic acid [30]. Similarly, Shah et al. noticed higher effectiveness of ceftriaxone (89.4%) against *Streptococcus viridians* compared to amoxicillin with clavulanic acid (68.1%) [33]. The same authors recommend replacing amoxicillin with ceftriaxone in the empirical management [31]. Unfortunately, although the sensitivity of aerobic Gram-positive cocci and *Porphyromonas gingivalis* to amoxycillin is high, there is substantial resistance present among other anaerobic bacteria, such as *Prevotella*, which are fundamental components of odontogenic infections [29].

Our study confirmed that metronidazole is successful against obligate anaerobes detected in odontogenic infections, such as *Veillonella*, *Fusobacterium*, and *Prevotella*. These findings are consistent with previous studies [31]. The combination of penicillin or amoxycillin with metronidazole seems to adequately cover the microbial flora of odontogenic infections and compensates for the limited activity of penicillin against anaerobic bacteria. Vavro et al. found a high level of metronidazole-resistant strains in maxillofacial infections, accounting for 41.4%. However, the same authors did not directly evaluate metronidazole resistance in anaerobic bacteria [34]. 

Moreover, our study revealed a high rate of resistance to macrolides such as erythromycin and high efficacy of fluoroquinolones such as ciprofloxacin and levofloxacin as well as aminoglycosides. However, their effectiveness is hard to assess due to limited data. Therefore, these results should be approached with caution. Serious side effects of these antibiotics appear to limit their use in odontogenic infections. Another prominent issue is the implementation of effective antibiotic therapy in specific infections. Although we detected *Schaalia* in only a few cases, our findings confirmed penicillin to be the most effective against these bacteria. Clindamycin and metronidazole should not be applied in actinomycosis.

Gram-negative opportunistic pathogens such as *Escherichia coli*, *Pseudomonas aeruginosa*, *Klebsiella pneumoniae*, *Klebsiella oxytoca*, *Acinetobacter ursingii*, *Serratia marcescens*, *Proteus mirabilis* and *Proteus hauseri* constituted a small percentage of isolated microorganisms in our study. According to previous studies, *Klebsiella pneumoniae* and *Pseudomonas aeruginosa* were more frequently detected in chronic periodontitis than in periapical infections. Moreover, they were mainly isolated from secondary spaces and phlegmonous infections. Judith et al. found that all these Gram-negative bacteria except for *Pseudomonas aeruginosa* were highly susceptible to amoxicillin–clavulanic acid [35]. Our findings show similarity to those obtained by Urechescu et al., who found that Gram-negative bacilli are resistant to β-lactams but susceptible to the third-generation cephalosporins, fluoroquinolones, and carbapenems [10]. Other studies found no differences in the distribution of *Escherichia coli*, *Pseudomonas aeruginosa*, and *Klebsiella pneumoniae* among microorganisms identified in dental practices and hospital maxillofacial departments. *Klebsiella* exhibited moderately high resistance to the second generation of cephalosporins and low resistance to the third generation of cephalosporins and fluoroquinolones [12].

The limitations of this study mainly arise from its retrospective design. Additionally, the diversity of patients as to age, causes of infection, and comorbidities could have a significant impact on the distribution of bacteria and their resistance to antibiotics. No correlation between the obtained bacterial results and clinical outcomes markedly limits data interpretation and makes it difficult to transfer them into clinical practice. We believe our results should be verified on a larger and more homogeneous group. Moreover, our study was conducted taking into account the patients who dwell in a single country. This does not allow drawing clear conclusions for the entire population.

Bacterial culturing, especially of obligate anaerobic bacteria, could also bring about some other study limitations. This is because the swab technique of sampling carries a higher risk of contamination than aspiration sampling. Previous self-medicated antibiotics could alter bacterial distribution in maxillofacial infections. Additionally, some of the data used in our study came from the final period of the then ongoing COVID-19 pandemic. It greatly impacted the accessibility of treatment for non-COVID-19 patients, including those with maxillofacial infections. Although the comparison of maxillofacial infections of the pre-pandemic and post-pandemic periods was not the purpose of this study, the pandemic itself could have modified the results obtained. It could have possibly been due to changes in hospitals’ functioning, restricted access to medical care, and the overuse of antibiotics. This impact may be permanent and should be verified in the following years. 

## 5. Conclusions

The polybacterial nature of maxillofacial infections, with predominant aerobic Gram-positive cocci, shows the need to implement antibiotics that effectively combat them or to combine two antibiotics to broaden their antibacterial spectrum and so as to fight aerobic and anaerobic bacteria. The polymicrobial profile of these infections, involving both aerobic and anaerobic bacteria, provides a pharmacological rationale for combined antibiotic therapy. High resistance of aerobic Gram-positive cocci (which dominate in maxillofacial infections) to clindamycin might suggest the need to reconsider clindamycin application in the empirical therapy of head and neck infections, with odontogenic infections in that number. Still, microbial resistance to clindamycin should be clearly distinguished from the potential clinical ineffectiveness of clindamycin in the treatment of maxillofacial infections. Cephalosporins could make good alternatives to penicillin in fighting maxillofacial infections, but this should be verified by further research and clinical practice. The culture-directed therapy plays a crucial role in reducing empirical clindamycin use. Metronidazole is the most effective antibiotic against obligate anaerobic bacteria. Given the presence of Gram-negative ESKAPE-class organisms, including *E. coli*, *Klebsiella*, *Serratia*, *Proteus*, *Acinetobacter* and *Pseudomonas* in a subset of cases, clinicians should maintain a low threshold for extended-spectrum empirical coverage in immunocompromised patients or those with severe or non-odontogenic infections.

## Figures and Tables

**Table 1:** Demographic and clinical characteristics of maxillofacial infection patients.

Parameter	Values
Number of individuals, *n*	224
Age, median (IQR)	39 (26.5)
Mean ± SD	41.6 ± 18.22
Ranges, years	15–91
Gender female/male, *n*	116/108
**Causes, *n*:**	
Odontogenic	206
Non-odontogenic	18
**Space involvement, *n***:	
Submandibular	94
Perimandibular	18
Buccal	35
Submental	13
Vestibular	20
Canine	15
Submasseterical	7
Pharyngeal	2
Sublingual	5
Pterygomandibular	5
Infratemporal	1
Others	10
**Accompanying systemic conditions, *n*:**	
Hypertension	38
Diabetes	8
Thyroid diseases	6
Obesity,	3
Depression,	3
Rheumatoid arthritis	3
Stroke	3
Other diseases (asthma, renal failure, psoriasis, alcoholism)	8
Pregnancy	2
**Antibiotic use, *n*:**	
Use of single antibiotic, *n*: amoxicillin, clindamycin, amoxicillin + clavulanic acid, metronidazole, ceftriaxone, cefixime, cefuroxime, ciprofloxacin, sulfametaxa-zole/trimethoprim	42
Use of two antibiotics, *n*: clindamycin, metronidazole, cefuroxime	180
Use of three antibiotics, *n*: tazobactam, metronidazole	2

IQR, interquartile ranges; %, percentage; *n*, number; SD, standard deviation.

**Table 2:** Detailed distribution of the identified bacterial cultures.

Bacteria	Number, *n* (%)
**Total**	400
**I.** **Bacillati**	292 (73%)
**1.** **Bacillota**	251 (62.75%)
**1.1.** **Bacilli**	229 (57.25%)
**1.1.1. Lactobacillales**	163 (40.75%)
1.1.1.1. **Streptococcus**:	156 (39%)
*Mitis/Oralis*,	64 (16%)
*Constellatus*,	16 (4%)
*Anginosus*,	22 (5.5%)
*Parasanguinis*,	17 (4.25%)
*Vestibularis*, *Sanguinis*,	6 (1.5%), 6 (1.5%)
*Intermedius*, *Salivarius*	5 (1.25%), 4 (1%)
*Gordonii*, *Cristatus*,	5 (1.25%), 4 (1%)
*Agalactiae*,	1 (0.25%)
*Identified as gr C*,	3 (0.75%)
*Identified as gr G*	1 (0.25%)
**1.1.1.2.** **Enterococcaceae**	
*Enterococcus faecalis*	4 (1%)
**1.1.1.3.** **Lactobacillaceae**	
*Limisilactobacillus oris*, *vaginalis*	1 (0.25%), 1 (0.25%)
*Lactobacillus plantarum*	1 (0.25%)
**1.1.2.** **Bacillales**	64 (16%)
**1.1.2.1.** **Staphylococcus:**	64 (16%)
*Epidermidis*,	48 (12%)
*Aureus*,	6 (1.5%)
*Hominis*, *Warneri*,	3 (0.75%), 2 (0.5%)
*Capitis*, *Auricularis*	1 (0.25%), 1 (0.25%)
*Haemolyticus*	1 (0.25%)
*Identified as coagulase-negative*	1 (0.25%)
**1.1.3.** **Caryophanales**	
**1.1.3.1.** **Gemellaceae**	
*Gemella morbillorum*, *heamolysant*	1 (0.25%), 1 (0.25%)
**1.2. Negativicutes**	16 (4%)
**1.2.1. Veillonellales**	16 (4%)
**1.2.1.1. Veillonellaceae:**	16 (4%)
*Veillonella parvula*, *atypica*	9 (2.25%), 2 (0.50%)
*Veillonella denticariosi*, *dispar*	1 (0.25%), 1 (0.25%)
*Veillonella*	1 (0.25%)
*Dialister pneumosintes*	2 (0.5%)
**1.3.** **Clostridia**	
**1.3.1.** **Tissierellales**	
**1.3.1.1.** **Peptoniphilaceae:**	
*Parvimonas micra*	3 (0.75%)
**1.4.** **Erysipelotrichia**	
**1.4.1.** **Erysipelotrichales**	
**1.4.1.1.** **Coprobacillaceae:**	
*Eggertella catenaformis*	2 (0.5%)
**1.4.1.2.** **Erysipelotrichaceae:**	
*Solobacterium moorei*	1 (0.25%)
**2.** **Actinomycetota**	40 (10%)
**2.1. Actinomycetes**	
**2.1.1. Micrococcales**	
**2.1.1.1. Micrococcaceae:**	
*Rothia mucilaginosa*	5 (1.25%)
*Rothia dentocariosa*, *kristinae*	4, 1 (0.25%)
**2.1.2. Mycobacteriales**	
**2.1.2.1. Corynebacteriaceae**	
*Corynobacterium falseni*	1 (0.25%)
**2.2. Actinomycetia**	
**2.2.1. Actinomycetales**	
**2.2.1.1. Actinomycetaceae:**	
*Schaalia odontolytica*, *meyeri*	7, 1 (0.25%)
**2.2.1.2. Propionibacteriaceae:** *Cutibacterium acnes*	9
**2.2.2. Bifidobacteriales**	
**2.2.2.1. Bifidobacteriaceae:**	
*Bifidobacterium dentium*	1 (0.25%)
*Parascardovia denticolens*	1 (0.25%)
**2.3.** **Coriobacteriia**	
**2.3.1.** **Coriobacteriales**	
**2.3.1.1.** **Atopobiaceae:**	
*Lancefieldella parvula*, *rimae*	5, 1 (0.25%)
*Lancefieldella*	1 (0.25%)
*Olsenella uli*	1 (0.25%)
**2.3.2.** **Eggerthellaceae:**	
*Slackia exigua*	2 (0.5%)
**II.** **Pseudomonadati**	93 (23.25%)
**1.** **Bacteroidota**	64 (16%)
**1.1.** **Bacteroidia**	57 (14.25%)
**1.1.1.** **Bacteroidales**	57 (14.25%)
**1.1.1.1.** **Prevotellaceae:**	56 (14%)
*Prevotella buccae*, *denticola*	14 (3.5%), 11 (2.75%)
*Prevotella melaninogenica*, *baroniae*	7 (1.75%), 4 (1%),
*Prevotella species*, *nigrescens*	3 (0.75%), 4 (1%),
*Prevotella oralis*, *intermedia*	5 (1.25%), 3 (0.75%)
*Prevotella salive*, *jejuni*,	1 (0.25%), 1 (0.25%)
*Prevotella histicola*, *maculosa*	1 (0.25%), 1 (0.25%)
*Alloprevotella rava*	1 (0.25%)
**1.1.1.2.** **Bacteroidaceae:**	
*Bacteroides pyogenes*	1 (0.25%)
**1.2.** **Flavobacteriia**	
**1.2.1.** **Flavobacteriales**	
**1.2.1.1.** **Flavobacteriaceae:**	
*Capnocytophaga spuntigea*, *ochracea*	4 (1%), 2 (0.5%)
*Capnocytophaga gingivalis*	1 (0.25%)
**2.** **Pseudomonadota**	29 (7.25%)
**2.1.** **Gammaproteobacterie**	23 (5.75%)
**2.1.1.** **Enterobacterales**	15 (3.75%)
**2.1.1.1.** **Enterobacteriaceae:**	13 3.25%)
*Escherichia coli*	5 (1.25%)
*Enterobacter hormaechei*, *bugandensis*	2 (0.5%), 1 (0.25%)
*Citrobacter amalonaticus*	1 (0.25%)
*Racultella ornithinolytica*	1 (0.25%)
*Klebsiella pneumoniae*, *oxytoca*	3 (0.75%), 1 (0.25%)
**2.1.1.2.** **Morganellaceae:**	
*Proteus mirabilis*, *hauseri*	1 (0.25%), 1 (0.25%)
**2.1.1.3.** **Yersiniaceae:**	
*Serratia marcescens*	1 (0.25%)
**2.1.2.** **Pseudomonadales**	5 (1.25%)
**2.1.2.1.** **Pseudomonadaceae:**	5 (1.25%)
*Pseudomonas aeruginosa*	2 (0.5%)
*Pseudomonas fluorescens*, *oryzihabitans*, *rhodesiae*	1 (0.25%), 1 (0.25%), 1 (0.25%)
**2.1.2.2.** **Moraxellaceae**	
*Acinetobacter ursingii*	1 (0.25%)
**2.1.3.** **Pasteurellales**	
**2.1.3.1.** **Pasteurellaceae:**	
*Haemophilus parainfluenzae*	1 (0.25%)
*Aggregatibacter aphrophilus*	1 (0.25%)
**2.2.** **β-proteobacteria**	4 (1%)
**2.2.1.** **Neisseriales**	4 (1%)
**2.2.1.1.** **Neisseriaceae:**	
*Eikenella corrodens*	4 (1%)
**2.3.** **Proteobacteria:**	
*Pantoea agglomerans*	1 (0.25%)
**III.** **Fusobacteriati**	16 (4%)
**1.** **Fusobacteriota**	16 (4%)
**1.1.** **Fusobacteriia**	
**1.1.1.** **Fusobacteriales**	
**1.1.1.1.** **Fusobacteriaceae:**	16 (4%)
*Fusobacterium nucleatum*	11 (2.75%)
*Fusobacterium necrophorum*	1 (0.25%)
*Fusobacterium periodonticum*	2 (0.5%)
*Fusobacterium*	1 (0.25%)
*Fusobacterium naviforme*	1 (0.25%)

%, percentage, *n*, number.

**Table 3:** Antibiotic resistance among aerobic and facultative anaerobic bacterial species.

Aerobic and Facultative Anaerobic Bacterial Species	Antibiotics	Resistance (%)
*Streptococcus*:		
	Amoxicillin/Ampicillin *n* = 141	19.85
	Other penicillin *n* = 148	18.91
	Ceftriaxone *n* = 143	5.59
	Cefuroxime *n* = 129	8.52
	Cefazolin *n* = 77	9.09
	Clindamycin *n* = 143	51.74
	Vancomycin *n* = 39	0
*Staphylococcus*:		
	Clindamycin *n* = 57	47.36
	Vancomycin *n* = 37	0
	Gentamycin *n* = 56	5.35
	Sulfamethoxazole/Trimethoprim *n* = 57	5.26
	Ciprofloxacin *n* = 49	8.16
	Cloxacillin *n* = 56	17.85
	Erythromycin *n* = 53	52.83
	Tetracycline *n* = 47	29.78
	Levofloxacin *n* = 49	8.16
*Enterococcus*:		
	Vancomycin *n* = 4	0
	Gentamycin *n* = 4	25.00
**Gram-positive cocci**	Other penicillin *n* = 149	12.38
	Amoxicillin/Ampicillin *n* = 145	12.38
	Ceftriaxone *n* = 144	3.53
	Cefuroxime *n* = 129	4.86
	Cefazolin *n* = 82	3.09
	Clindamycin *n* = 200	44.69
	Gentamycin *n* = 60	1.76
	Sulfamethoxazole/Trimethoprim *n* = 63	1.32
	Ciprofloxacin *n* = 49	1.76
	Cloxacillin *n* = 56	4.42
	Erythromycin *n* = 59	12.83
	Tetracycline *n* = 53	7.07
	Levofloxacin *n* = 52	1.76
**Bacilli:**		
**Gram-negative bacilli:**	Other penicillin *n* = 7	14.70
	Amoxicillin/Ampicillin *n* = 2	2.94
	Amoxicillin + clavulanic acid *n* = 14	14.70
	Clindamycin *n* = 8	14.70
	Metronidazole *n* = 7	5.88
	Meropenem *n* = 24	2.94
	Sulfamethoxazole/Trimethoprim *n* = 15	8.82
	Ciprofloxacin *n* = 21	8.82
*Pseudomonas*:	Cefazolin *n* = 5	0
	Gentamycin *n* = 5	0
	Meropenem *n* = 5	0
	Tazobactam *n* = 5	0
	Ciprofloxacin *n* = 5	0

%, percentage; *n*, number of isolates tested for that antibiotic.

**Table 4:** Antibiotic resistance of anaerobic bacteria.

Bacterial Species	Antibiotics	Resistance (%)
*Prevotella*:		
	Amoxicillin/Ampicillin *n* = 31	64.51
	Other penicillin *n* = 48	62.50
	Amoxicillin + clavulanic acid *n* = 34	50
	Clindamycin *n* = 47	59.57
	Metronidazole *n* = 47	4.25
	Meropenem *n* = 39	0
	Tazobactam *n* = 45	4.44
	Imipenem *n* = 55	0
*Fusobacterium*:		
	Other penicillin *n* = 12	8.33
	Clindamycin *n* = 11	9.09
	Metronidazole *n* = 11	0
*Veillonella*:		
	Other penicillin *n* = 10	60.00
	Amoxicillin + clavulanic acid *n* = 9	22.22
	Clindamycin *n* = 12	8.33
	Metronidazole *n* = 10	0
*Schaalia*:		
	Other penicillin *n* = 6	0
	Clindamycin *n* = 6	33.33
	Metronidazole *n* = 6	60
*Lancefieldella*:		
	Other penicillin *n* = 6	0
	Clindamycin *n* = 6	50
	Metronidazole *n* = 6	0
**Gram-negative cocci**	Other penicillin *n* = 10	42.85
	Amoxicillin + clavulanic acid *n* = 9	14.28
	Clindamycin *n* = 12	7.14
**Gram-positive cocci**	Other penicillin *n* = 4	25.00
	Clindamycin *n* = 4	75.00
	Metronidazole *n* = 4	25.00
**Bacilli:**		
**Gram-negative bacilli**	Amoxicillin/Ampicillin *n* = 33	26.66
	Other penicillin *n* = 60	41.33
	Amoxicillin + clavulanic acid *n* = 39	22.66
	Clindamycin *n* = 60	38.66
	Metronidazole *n* = 60	2.66
	Tazobactam *n* = 52	2.66
**Gram-positive bacilli**	Clindamycin *n* = 26	19.35
	Metronidazole *n* = 19	25.80

%, percentage; *n*, number of isolates tested for that antibiotic.

**Table 5:** Comparing antibiotic resistance between aerobic or facultative anaerobic bacteria and obligate anaerobic bacteria.

Antibiotic	Antibiotic Resistance in Aerobic and Facultative Anaerobic Bacteria (%)	Antibiotic Resistance in Obligate Anaerobic Bacteria (%)	*p* Value
Other penicillin *n* = 270	21.30	37.62	**0.003 ^1^**
Amoxicillin/Ampicillin *n* = 191	19.46	47.62	**˂** **0.001 ^1^**
Amoxicillin + clavulanic acid *n* = 78	33.33	30.16	1.000 ^2^
Ceftriaxone *n* = 180	5.20	0	1.000 ^2^
Cefuroxime *n* = 152	7.43	0	1.000 ^2^
Cefazolin *n* = 99	7.14	0	1.000 ^2^
Clindamycin *n* = 314	50.0	38.24	0.050 ^1^
Metronidazole *n* = 105	20.0	11.58	0.608 ^2^
Meropenem *n* = 79	3.70	0	0.341 ^2^
Tazobactam *n* = 89	0	3.08	1.000 ^1^

%, percentage; *n*, number of isolates tested for that antibiotic; ^1^, Chi^2^ Pearson’s test; ^2^, Fisher’s exact test.

**Table 6:** Comparing antibiotic resistance between types of bacteria.

	Types of Bacteria			
Antibiotics	Firmicutes	Fusobacteriota	Proteobacteria	*p* Values
Amoxicillin/Ampicillin *n* = 148	19.18%	0%	0%	˂1 ^1^
Other penicillin *n* = 163	19.21%	8.33%		0.697 ^2^
Amoxicillin + clavulanic acid *n* = 14	0%	0%	27.27%	˂1 ^1^
Ceftriaxone *n* = 159	5.59%		0%	˂1 ^2^
Cefuroxime *n* = 143	8.53%		0%	0.601 ^2^
Cefazolin *n* = 95	8.54%		0%	0.588 ^2^
Clindamycin *n* = 215	49.75%	9.09%	100%	**0.010 ^1^**
Gentamycin *n* = 78	6.67%		0%	0.568 ^2^
Sulfamethoxazole/Trimethoprim *n* = 75	4.76%		33.33%	**0.010 ^2^**
Ciprofloxacin *n* = 67	8.16%		16.67%	0.375 ^1^
Levofloxacin *n* = 58	7.69%		0%	˂1 ^1^

^1^, Fisher–Freeman–Halton’s test; ^2^, Fisher’s exact test; *n*, number of isolates tested for that antibiotic.

**Table 7:** Comparing antibiotic resistance between phylum of bacteria.

	Phylum of Bacteria				
Antibiotics	Bacillota	Actinomycetota	Bacteroidota	Pseudomonadota	*p* Value
Amoxicillin/Ampicillin *n* = 186	18.79%	0%	63.64%	0%	**˂0.001 ^1^**
Other penicillin *n* = 250	21.43%	7.41%	63.64%		**˂0.001 ^2^**
Amoxicillin + clavulanic acid *n* = 67	13.33%	0%	47.22%	38.46%	0.073 ^1^
Ceftriaxone *n* = 173	5.56%	12.50%		0%	0.292 ^1^
Cefuroxime *n* = 148	8.53%	0%		0%	0.408 ^1^
Cefazolin *n* = 99	8.54%			0%	0.600 ^3^
Clindamycin *n* = 294	47.75%	29.41%	59.26%	100%	0.083 ^1^
Metronidazole *n* = 91	9.09%	33.33%	7.41%		**0.027 ^1^**
Meropenem *n* = 70	0%	0%	2.38%	0%	˂1 ^1^
Tazobactam *n* = 79	0%	0%	4.17%	0%	˂1 ^1^
Gentamycin *n* = 80	6.67%			0%	0.567 ^3^
Sulfamethoxazole/Trimethoprim *n* = 80	4.76%	0%		25.00%	0.077 ^1^
Ciprofloxacin *n* = 73	8.16%	0%		13.04%	0.703 ^1^
Levofloxacin *n* = 58	7.69%			0%	˂1 ^3^

^1^, Fisher–Freeman–Halton’s test; ^2^, Chi^2^ Pearson’s test; ^3^, Fisher’s exact test; *n*, number of isolates tested for that antibiotic.

## Data Availability

The original contributions presented in this study are included in the article. Further inquiries can be directed to the corresponding author.
